# An auditory processing advantage enables communication in less complex social settings: Signs of an extreme female brain in children and adolescents being assessed for Autism Spectrum Disorders

**DOI:** 10.3389/fpsyg.2022.1068001

**Published:** 2023-01-13

**Authors:** Sofia Åkerlund, Anders Håkansson, Emma Claesdotter-Knutsson

**Affiliations:** Department of Clinical Sciences, Faculty of Medicine, Lund University, Lund, Sweden

**Keywords:** ASD, “gender differences”, “sensory processing”, “child and adolescent psychiatry”, auditory, “extreme female brain”, “processing advantage”

## Abstract

**Background:**

The underlying factors of the male predominance in Autism Spectrum Disorders (ASD) are largely unknown, although a female advantage in social communication has been pointed out as a potential factor. Recently, attention has been given to ASD as a sensory processing disorder, focusing on the audio-visual temporal processing paramount for the development of communication. In ASD, a deviant audio-visual processing has been noted, resulting in difficulties interpreting multisensory information. Typically Developed (TD) females have shown an enhanced language processing in unisensory situations compared to multisensory situations. We aim to find out whether such an advantage also can be seen in girls within the ASD population, and if so, is it related to social communication skills?

**Method:**

Forty children (IQ > 85), 20 females (mean age = 13.90 years, SD = 2.34) and 20 males (mean age = 12.15 years, SD = 2.83) triaged for an ASD assessment were recruited from a child and youth psychiatric clinic in Sweden. Using The Social Responsiveness Scale (SRS) we looked at associations with child performance on the Integrated Visual and Auditory Continuous Performance Test (IVA-2).

**Results:**

An auditory advantage in the female group was associated with less rated problems in social communications in unisensory processing whereas in multisensory processing an auditory dominance was associated with more rated problems in Social Awareness. In the male group, a visual dominance was associated with more rated problems in Social Rigidity.

**Conclusion:**

A female unisensory processing advantage in ASD could very well be explaining the male domination in ASD. However, the social difficulties related to multisensory processing indicate that ASD females might be struggling as hard as males in more complex settings. Implications on the assessment procedure are discussed.

## Introduction

Autism Spectrum Disorder (ASD) is a set of heterogeneous neurodevelopmental conditions characterized by difficulties in social communication and restricted, repetitive behavior and interests [American Psychiatric Association (APA), 2013]. Historically, ASD was considered a male disorder, although the view has changed there is still a male dominance with a 3:1 ratio (Hull et al., 2017; Loomes et al., 2017; Chiarotti and Venerosi, 2020; Saito et al., 2020). The male predominance in ASD is far from being fully understood, however, since the domination is mostly prominent in the group of High Functioning patients, those with an Intelligence Quotient (IQ) of 75 and above, some mean it is insufficient theoretical knowledge and clinical insight about the ASD female profile that is a part of the explanation (Moseley et al., 2018; Young et al., 2018). Others mean the female double set of x-genes provides a biological protection from ASD (Baron-Cohen et al., 2011; Werling, 2016).

In terms of communication, there is support for both theories. A female superiority in language development is well established although the gender difference in the normal population is very small, larger differences have been seen in those on the 10th percentile and below, boys being twice as likely to be diagnosed with a language disorder (Wallentine, 2020). In support for there being a lack of knowledge of female ASD, there are studies showing autistic girls to generally be more positively rated by a novel conversation partner than autistic boys despite an equal level of autism severity (Rynkiewicz et al., 2016; Dean et al., 2017; Parish-Morris et al., 2017; Cola et al., 2020).

“Camouflaging” refers to the ability to meet social demands by mimicking behavior in a seemingly flexible way and is more common in ASD girls than boys. While the use of camouflaging strategies requires some understanding of social communication, the lack of flexibility typically leaves the individual exhausted and in risk of developing various depression- and anxiety-related disorders (Rynkiewicz et al., 2019). Camouflaging strategies are said to blur important distinctions and inadvertently contribute to misconstruing important clinical and eligibility decisions even though these children/adolescents are insightfully rated by those who know them well in contextually relevant situations (Hull et al., 2017; Young et al., 2018).

A verbal IQ of 70 and above has shown to be positively related to age of diagnosis in both genders when controlling for demographic factors. The association has shown to be far stronger for girls which means that being a female with good verbal skills will be putting you at risk for a delayed autism diagnosis (McDonnell et al., 2021) which, in turn, increases the risk of the patient developing more severe psychiatric symptoms (Rynkiewicz et al., 2019).

In the past few years, the view of ASD as a sensory processing disorder has received more attention (Robertson and Baron-Cohen, 2017; Posar and Visconti, 2018; Zhou et al., 2021), specifically the audio-visual temporal processing and its effect on language development (Ocak et al., 2018; Tanigawa et al., 2018; Jain et al., 2020; Meilleur et al., 2020), social abilities (Wallace and Stevenson, 2014), and to form a coherent perception of the world (De Niear et al., 2018). Sensory processing difficulties, defined as hyper- and hypo-sensitive responses to sensory information, are reported in more than 96% of children with ASD (Marco et al., 2011; Robertson and Baron-Cohen, 2017).

While sensory processing refers to the ability to detect, regulate, interpret, and respond to sensory stimuli (Dunn, 2001), temporal processing refers to the ability to integrate contemporary sensory inputs into an adequate global interpretation of the whole (Wallace and Stevenson, 2014). The Sensory Integration Theory (SIT; Ayres, 1979) states that the human development is strongly affected by the process and integration of sensory inputs. It differs between *Unisensory* and *Multisensory* processing, the first referring to the process of one or more stimulus from one sensory modality such as auditory *or* visual stimuli, whereas the second refers to the integration process of stimuli received from different modalities such as auditory *and* visual stimuli.

The Temporal Binding Window (TBW) is used to describe the period of time passing between the exposure of two stimuli in order for them to still be perceived as bind together. In terms of auditory and visual unisensory temporal processing, studies of ASD show varying results, some indicating that there is a larger TBW in people with autism whereas others indicate that it is smaller (Zhou et al., 2018; Meilleur et al., 2020). However, age seems to be affecting the results as adults with ASD showed to be better in visual discrimination than TD, indicating a smaller visual TBW than TD (Falter et al., 2012), whereas children with ASD showed no enhanced visual discrimination but rather an impaired auditory discrimination, indicating a larger auditory TBW than TD (Kwakye et al., 2011). However, both studies are largely dominated by male subjects, and neither considered gender as a factor.

Research of audio-visual temporal processing in ASD shows more consistent findings. A reduced audio-visual temporal acuity is well established (Shams et al., 2000; Alais and Burr, 2004; Zhou et al., 2021). Several studies show that the audio-visual TBE in children and youths with ASD is larger than in TD, however, the results are restricted to audio-visual speech stimuli (Woynaroski et al., 2013; Stevenson et al., 2014; Noel et al., 2018). One study showed no audio-visual temporal processing impairments in youth with ASD (de Boer-Schellekens et al., 2013); however, subjects in this study were young adults including adults and mainly of male gender which might have skewed the results. In a review article covering studies of temporal processing in ASD (Meilleur et al., 2020) lack of age as a developmental variable is noted. Still, it is concluded that the collected material points toward a delayed maturation of multiple sensory integration in children with ASD, since studies of adults with ASD show better audio-visual integration performance than studies with ASD children presented with the same task.

It is common for children with ASD to have one sensory modality that is superior, meaning that it responds faster or stronger than others (Bebko et al., 2006; Kwakye et al., 2011; Foss-Feig et al., 2017; Balasco et al., 2020). A superior modality has shown to dominate the perception, blocking out information from other sources, leaving the person with a lack of information when trying to grasp the full concept of a situation (Meilleur et al., 2020).

Several studies show ASD children to be superior in visual acuity (Jolliffe and Baron-Cohen, 1997; Joseph et al., 2009; Kaldy et al., 2016). Considering that 80% of included participants in ASD studies from the past 10 years are of male gender and that studies rarely separate between gender (Feldman et al., 2018), we should be cautious accepting results stating that autistic children show certain characteristics. However, the belief of ASD females showing cognitive similarities to a male cognitive style is supported in studies of brain structure and function (Lai et al., 2013) as well as by theories meaning ASD should be seen as an “extreme male brain” (Asperger and Frith, 1991; Baron-Cohen et al., 2011). However, we must bear in mind that all studies including patients already diagnosed with autism will present us with biased data. The screening instrument for autism is based on research mostly made on males; hence, the children passing for a diagnosis will also be matching with the male phenotype of autism whether their biological gender is male or female.

According to the empathizing-systemizing (E-S) theory of psychological sex differences, male brains show a stronger systemizing ability, whereas females show a stronger ability of empathizing. Systemizing is defined as the drive to analyze and predict the behavior of a system, whereas empathizing is defined as the drive to analyze and predict other people’s mental states (Baron-Cohen, 2003). The E-S theory differentiates between individual brain types by measuring the dimensions of empathizing and systemizing resulting in five different types. Those having an equal amount of empathizing and systemizing are categorized as *Type B* (E = S). *Type E* show a stronger ability of empathizing than systemizing (E > S) and the reversed situation is represented by *Type S* (E < S). *Extreme Type E* represent those with an extreme ability for empathizing and is more common in females, whereas *Extreme Type S* represent those with an extreme ability for systemizing and is more common in males (Greenberg et al., 2018). When people diagnosed with ASD are categorized in accordance with the E-S brain types, results indicate *Extreme Type E* is highly unusual in both genders (Greenberg et al., 2018) which seems quite logical considering the biased assessment procedure of ASD mentioned above. In another study though, Floris et al. showed that the “male brain syndrome” can only be said to describe a subgroup of the ASD population (Floris et al., 2018) which is supported by DiCriscio and Troiani who published a study showing that an enhanced visual ability is only associated with ASD symptoms in ASD males (DiCriscio and Troiani, 2017).

Rather than relying on old ASD research, perhaps, it is more reasonable to turn our attention toward the normal population for cues as to how we should understand and investigate female ASD.

While TD adult males have shown a visual acuity processing advantage, TD females have shown an auditory acuity processing advantage (McGivern et al., 2019; Siedlecki et al., 2019; Thornton et al., 2019). If the male phenotype of ASD is having an *“extreme male brain”* would it not be reasonable to assume that a female phenotype of autism would involve an *“extreme female brain”?*

An extreme female brain would then be characterized by a superior auditory acuity. Auditory acuity has previously been associated with speech comprehension as well as the development of language and social communication (Lee et al., 2018; Ayasse et al., 2019). In a study from 2017, a better signal-detection ability was seen in people with ASD using camouflaging strategies (Lai et al., 2017; Parish-Morris et al., 2017).

Speech comprehension requires the use of both visual and auditory information processes (King et al., 2019). TD males have shown to be more lateralized in their language processing than TD females, meaning that they favor the left hemisphere where most of the auditory comprehension take place. Females, on the other hand, use bilateral language processes, meaning that they rely on both auditory and visual information to a higher degree than males (Burman et al., 2008; Koles et al., 2010; Ross et al., 2015). As mentioned above, a superior sensory modality will block out information from the other modality; hence, a superior visual ability will be blocking out auditory information whereas a superior auditory ability will be blocking out visual information. Mainly relying on auditory information, the male lateralized language process will be highly vulnerable when faced with a superior visual ability. The bilateral language processing seen in females will be more robust, providing an alternative processing rout when faced with a visual or auditory superior ability. However, in a more complex setting where multisensory integration is required, females will be as vulnerable as males when faced with a superior auditory or visual ability. A superior auditory ability will block out visual information needed to understand the full complexity of a setting.

In a review article from 2020, neurobiological sex differences in language processing are rejected due to the lack of consensus found between results from previous studies (Sato, 2020). However, the studies included in the reviews differ in many ways from each other, some using word generation as a measure, others counting, picture naming, or lip reading, which is not a fair comparison since neural organization of language has shown to be task dependent (Hickok and Poeppel, 2007).

The theory of Social Motivation discriminates between less complex social settings, requiring unisensory information to be processed, and more complex social behavior requiring multisensory integration to be processed (Tamir and Hughes, 2018). In a study from 2021, adult TD women showed an enhanced emotional identification in unisensory processing tasks compared to TD males, whereas in multisensory integration tasks, no gender differences were seen (Lin et al., 2021).

Hypothesizing that female children and youths with ASD have an extreme female brain, it could provide us with an explanation as to why ASD females on a group level, are better social performers in some settings, while still experiencing difficulties in others.

The aim of our study is to look at the auditory and visual processing in boys and girls between seven and 17 years of age. By including measurements representing auditory and visual unisensory and multisensory processing we aimed to look for potential associations with parental ratings of the Social Responsiveness Scale. Since 40%–70% of the ASD population show a comorbidity with Attention Deficit Hyperactivity Disorder (ADHD; Antshel and Russo, 2019), defined by difficulties with inattention and hyperactivity with an onset before the age of 10 [American Psychiatric Association (APA), 2013], we included an ADHD rating scale in order to control for hyperactivity and inattention. As to our knowledge, no previous studies have been looking into this before.

The Integrated Visual and Auditory Continuous Performance Test (IVA CPT; Sandford and Turner, 2000) is a continuous performance task administered using a computerized format. IVA measures various attention-related components in terms of both visual and auditory stimuli. Social Responsiveness Scale (SRS; Constantino and Gruber, 2012) is one of the most used standardized assessments of ASD both internationally and in Sweden (Zander, 2021). It surveys the core symptoms of autistic traits in social communication (Constantino et al., 2003). The Swanson, Nolan, and Pelham scale (SNAP-IV) is a widely used rating scale of ADHD (Hall et al., 2020). It gives scores within the core symptoms of ADHD, i.e., inattentiveness and hyperactivity as well as symptoms of Oppositional Defiance Disorder (ODD; Swanson et al., 2012).

We hypothesized that a female auditory processing advantage would be seen in unisensory processing by associations between a better auditory ability and less rated problems in SRS whereas in the male group no associations between a superior auditory or visual ability and less rated problems in SRS would be seen. In the multisensory IVA measurements, we assumed the females would show associations between an auditory dominance and more rated problems within SRS whereas in the male group a visual dominance would be associated with more rated problems within SRS.

## Materials and methods

### Participants

To avoid as many assessment biases as possible, all children triaged for an ASD assessment during the year of 2017 were invited to participate in the study whether they later receive an autism diagnose or not. Fifty-seven children between the ages of 7–17 (29 females mean age 12.97 years, SD 3.168 and 28 males, mean age 11.71 years, SD 2.904) were recruited from the child and adolescent psychiatric out-patient clinic in Eslöv, Sweden (Table 1). The children were from the same socioeconomic area, the communities of Eslöv, Höör, and Hörby where the median wage is around 74% of the Swedish median wage and has an unemployment rate of 20% compared to 9.4% for all of Sweden (Statistiska Centralbyrån, 2021).

**Table 1:** Group statistics.

	Females	Males
Recruited	29	28
Average age (SD)	12.97 (3.17)	11.71 (2.90)
Excluded	8	7
Outliers	1	1
Remained	20	20
Average age^a^ (SD)	13.90 (2.34)	12.15 (2.83)
WISC-V Intelligence quotient	>85	>85

SD, Standard deviation.

a Participants included in the study.

The children were triaged either through a screening procedure done by clinical psychiatric nurses using the structured Brief Child and Family Phone Interview (BCFPI; Boyle et al., 2009; Cunningham et al., 2009) or by referral from other clinical professionals. Four girls and seven boys already had a diagnosis of ADHD and were included in the study unmedicated. The remaining 46 children had no prior neuropsychiatric diagnosis. To be included in the study a verbal as well as fluid intelligence quotient of 85 and above was required which was measured with the Wechsler Intelligence Scale for Children (WISC-V; Wechsler, 2003). Exclusion criteria were a diagnosis of any hearing disabilities including tinnitus, difficulties communicating in Swedish, and any form of substance abuse. Nine children were excluded due to not passing the IVA validity scales. Four children interrupted and did not want to continue the IVA testing and were therefore excluded. Two children were excluded when screening for outliers within the IVA-results and another two were excluded due to parental assessments not being completed. The remaining participants were 20 females (mean age 13.90 yrs., SD 2.34) and 20 males (mean age 12.15 yrs., SD 2.83). The females had a mean score of 69.8 in the parental ratings of Social Cognition and a mean score of 66.6 in the ratings of Social Communication, whereas the males had a mean score of 64.8 in Social Cognition and 64.3 in Social Communication, indicating females were rated as having higher problems in both Social Cognition and Social Communication than the males. A written informed consent was obtained from all children and their guardians. The study was approved by the regional ethics committee in Lund (Dnr: 2016/964).

### Tests

#### IVA-2 CPT

IVA-2 (Sandford and Turner, 2000) is a computerized continuous performance test integrating visual and auditory sensory processes. The visual stimuli are presented on the computer screen, while the auditory stimuli are presented *via* headphones equipped with ear cushions. The output consists of 20 different basic measurements, each providing a combined visual–auditory measure as well as independent measurements of auditory and visual EF. The basic measurements are also used in the construct of different scales. The four primary scales are attention, sustained attention, response control, and symptomatic problems. Eight subscales provide a combined auditory-visual processing score as well as separate processing scores for auditory and visual function. The Validity Scales control for lack of comprehension, unwillingness to participate, or other misconduct behavior. The unisensory measurements used in this study were chosen to represent aspects in ASD that are either enhanced (Acuity) or dysfunction (Focus, Elasticity). A more complex unisensory measurement in the form of two scales was also used to measure high- and low-demanding tasks. Finally, we also used the auditory–visual difference score from the two extreme measurements of Focus as well as Scale of Agility as a measurement of multisensory processing (Table 2). The IVA-2 profile is summarized quantitatively through standard scores that are familiar to most clinical practitioners. An IVA-testing not passing the validity scales shows no results. Test time: 15 min.

**Table 2:** IVA-measurements used.

	Measurements		
Unisensory processing	Elasticity	Acuity	Focus
Auditory	×	×	×
Visual	×	×	×
Complex unisensory processing	Scale of competence (high-demanding processing)	Scale of maintenance (low-demanding processing)	
Auditory	×	×	
Visual	×	×	
Multisensory processing	Scale of focus	Scale of agility	
A/V diff.	×	×	
A/V diff. Inv.	×	×	

#### The Swanson, Nolan, and Pelham scale

The Swanson, Nolan, and Pelham scale (SNAP-IV; Swanson et al., 2012) provides measurements of basic ADHD symptoms of inattention, hyperactivity as well as Oppositional Defiant Disorder (ODD). The scale was included since the same symptoms are common in patients with autism. The scale consists of 26 questions divided into three different groups, the first nine being related to inattention, the following nine to impulsivity/hyperactivity, and the remaining eight to ODD. The rating span 0–3 corresponds to the child showing a certain behavior “not at all,” “just a little,” “quite a bit” and “very much.” An average score for each subscale is calculated and used as a measurement, hence ranging from 0.0–3.0. A score above 1.0 indicates deviances. Test time: 10 min.

#### Social responsiveness scale

The Social Responsiveness Scale (SRS-2; Constantino and Gruber, 2012) measures behavior of a child or adolescent between the ages of 4 and 18, serving as one index of severity of social deficits in ASD. It consists of 65 questions (17 of which are reverse scored) divided into five subscales measuring Social Cognition (ability to interpret social cues once they are picked up), Social Communication (ability to be motoric expressive in social communication), Social Awareness (ability to pick up on social cues), Social Motivation (motivation to engage in social-interpretational behavior), and Social Rigidity (stereotypical behaviors and restricted interests; Constantino and Gruber, 2012). The rating span 0 to 3 represents, in corresponding order: Not true, somewhat true, often true, and always true. The score is compared to a norm curve and the final measurement used is a t-scale score. A t-score above 60 raises suspicions the child/adolescent may be at risk or have signature features consistent with ASD. Test time: 30 min.

### Procedure

The IVA-tests were administered by trained staff. Participants were seated on a comfortable chair, adjusted to give the child a comfortable and easy-to-reach position. The participant was presented with the auditory stimuli through tight-fit headphones to reduce ambient noise that might be needless distracting. The test room was empty, and the windows were covered up to shut out possible disturbing visual stimuli outside. The participants were presented with a session of 2 × 1 min of responding to auditory and visual stimuli one at a time. After that, a training session of 1.5 min started where the child got to practice responding to both kinds of stimuli in a random order. The test starts when the computer has registered a proper response pattern in the practice part. The main test consists of either a written number 1 or 2 on the computer screen or a voice reading “one” or “two.” The computer voice tells the participants when they are to click on the mouse and when they are not to. The parental rating scales as well as a reply envelope were given to the parents at the first assessment visit to the clinic.

### Data analysis

Analyses were done using the Statistical Package for Social Sciences (SPSS; IBM Corp, 2019) using a significance level of 0.05 in all tests. Before further analyzing, the IVA-data were screened for unusual cases above 3 x the interquartile range. The descriptive statistics were used for calculating group mean scores and standard deviations. The Kolmogorov–Smirnov test was used to test for normal distribution. The independent samples *t*-test was used when calculating group mean differences in age. Levene’s test of variance was used to explore the homogeneity of variances. An analysis of covariance (ANCOVA) test was used to examine group mean differences within the measurements from the parental scales and the *a priori* chosen IVA measurements, controlling for the covariate of Age. The Bonferroni correction was used to adjust for multiple correlations. The Pearson’s correlation test was used when determining relationships between the parental ratings and the selected IVA-measurements using age as a control variable.

As measures for the unisensory processing, we used the independent measures of auditory and visual Focus, Acuity, and Elasticity as well as two more complex unisensory measures in the form of scales, each providing a combined measure of three different aspects from the same modality. The Scale of Competence represented high-demanding tasks whereas the Scale of Maintenance represented low-demanding tasks. As a measure of multisensory processing, we used the difference score between auditory and visual performance in Focus, an individual measure and in the Scale of Agility. A positive difference score indicates an auditory dominance whereas a negative score indicates a visual dominance (Table 2). The IVA-scores were correlated with each of the parental SRS assessment variables. All the correlations were calculated using Pearson rho. The results are presented in r-values between −1 to +1. A positive value indicates a positive correlation, and a negative value indicates an inverse relation. A value around 0 indicates no correlation.

## Results

Two patients showed extreme outliers, above the 3 × inter-quarter range (IQR) in several of the IVA-measurements, one male and one female. Both were excluded from the study. Another outlier was detected in the female group within Auditory Acuity showing an extreme low result. The subject was kept in the study but left out from affected analyses, hence the participant was excluded from the analyses including Auditory Acuity and Auditory Scale of Maintenance. The *t-*test used for equality of means in age showed a significant result (Sig. 2-tailed 0.040) with the females having a 1.75 year higher mean age than the males. Levene’s test of Equality of Error Variances turned out positive in the cases of Social Rigidity (sig = 0.006), Auditory Acuity (sig = 0.011), and Auditory Scale of Maintenance (sig ≤ 0.001), the females showing a higher variance than the males in the ratings of Social Rigidity and the males showing a higher variance in the measurements of auditory Acuity and auditory Maintenance (Table 3).

**Table 3:** Group statistic of the estimated mean score of the IVA-measurements.

Dependent variable	Gender	*N*	Estimated mean	Standard estimated error
Social awareness	Females	20	56.62^a^	3.223
	Males	20	60.23^a^	3.223
Social cognition	Females	20	70.058^a^	3.664
	Males	20	64.492^a^	3.664
Social motivation	Females	20	72.948^a^	3.069
	Males	20	65.952^a^	3.069
Social communication	Females	20	67.258^a^	3.399
	Males	20	63.642^a^	3.399
Social rigidity	Females	20	75.890^a^	3.958
	Males	20	65.710^a^	3.958
ODD	Females	20	1.341^a^	0.172
	Males	20	1.051^a^	0.172
Inattention	Females	20	1.69^a^	0.165
	Males	20	1.700^a^	0.165
Hyperactivity	Females	20	1.052^a^	0.174
	Males	20	1.183^a^	0.174
Auditory acuity	Females	19	95.932^b^	6.361
	Males	20	75.438^b^	6.129
Visual acuity	Females	20	88.265^a^	6.414
	Males	20	80.235^a^	6.414
Auditory elasticity	Females	20	87.797^a^	5.727
	Males	20	81.903^a^	5.727
Visual elasticity	Females	20	79.758^c^	7.314
	Males	19	81.292^c^	7.314
Auditory focus	Females	20	84.210^a^	4.169
	Males	20	78.840^a^	4.169
Visual focus	Females	20	93.658^a^	4.700
	Males	20	86.292^a^	4.700
Auditory maintenance	Females	19	95.932^b^	6.421
	Males	20	71.164^b^	6.250
Visual maintenance	Females	20	81.942^a^	7.545
	Males	20	62.408^a^	7.545
Auditory competence	Females	20	81.757^a^	6.616
	Males	20	77.493^a^	6.616
Visual competence	Females	20	76.706^a^	6.194
	Males	20	75.494^a^	6.194

a Covariates appearing in the model are evaluated at the following values: Age = 13.0250.

b Covariates appearing in the model are evaluated at the following values: Age = 12.9744.

c Covariates appearing in the model are evaluated at the following values: Age = 10.1026.

The ANCOVA showed significant differences between the group means in the measure of Auditory Maintenance (Table 4).

**Table 4:** Levene’s test of equality of variances as well as the ANCOVA for variables showing significant differences between the groups.

				Levene’s test of equality of error variance				Pairwise comparisons		
Dependent variable	Gender (*N*)	Mean	Std. deviation	F	Sig.	Gender		Estimated mean difference (I-J)	Std. error	Sig.^c^
						I	J			
Social rigidity	Females (20)	74.8500	21.25107	8.550	0.006^**^	Female	Male	5.566^a^	5.753	0.085
	Males (20)	66.7500	11.96431			Male	Female	−5.566^a^	5.753	0.085
Auditory acuity	Females (19)	94.3684	16.00420	7.198	0.011^*^	Female	Male	16.416^b^	9.104	0.080
	Males (20)	73.0500	35.34711			Male	Female	−16.416 ^b^	9.104	0.080
Auditory scale of maintenance	Females (19)	96.8947	13.96382	14.339	<0.001^***^	Female	Male	24.768^b^	9.190	0.011^*^
	Males (20)	70.2500	35.17606			Male	Female	−24.768^b^	9.190	0.011^*^

* The mean difference is significant at the 0.05 level.

** The mean difference is significant at the 0.01 level.

*** The mean difference is significant at the 0.001 level.

a Covariates appearing in the model are evaluated at the following values: Age = 13.0250.

b Covariates appearing in the model are evaluated at the following values: Age = 12.9744.

c Adjustments for multiple comparisons: Bonferroni.

**Figure 1:**
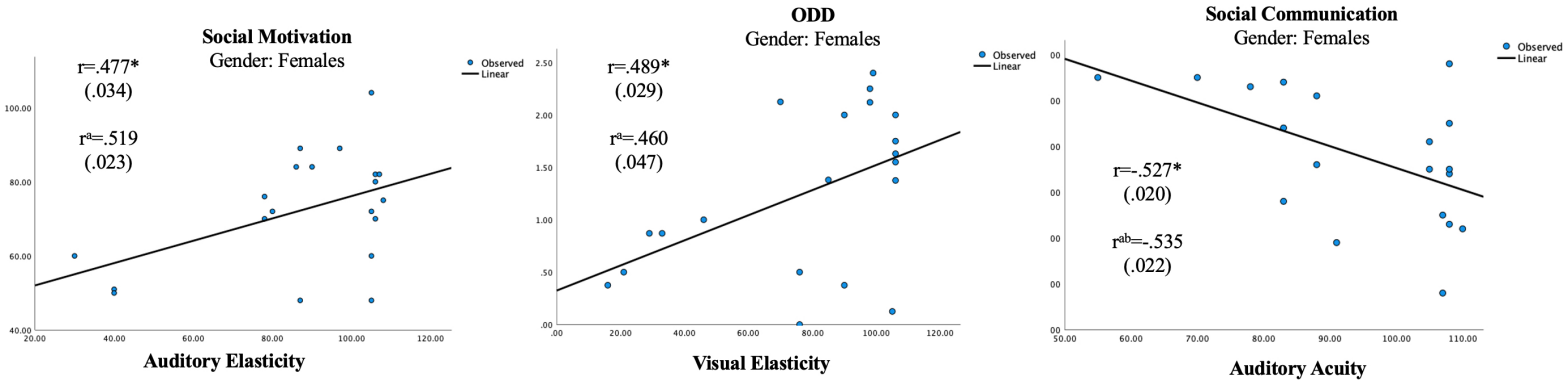
Significant correlations in unisensory processing in the female group. ^a^Using age as a control variable. ^b^Accounted for 19 subjects. ^*^p < 0.05.

**Figure 2:**
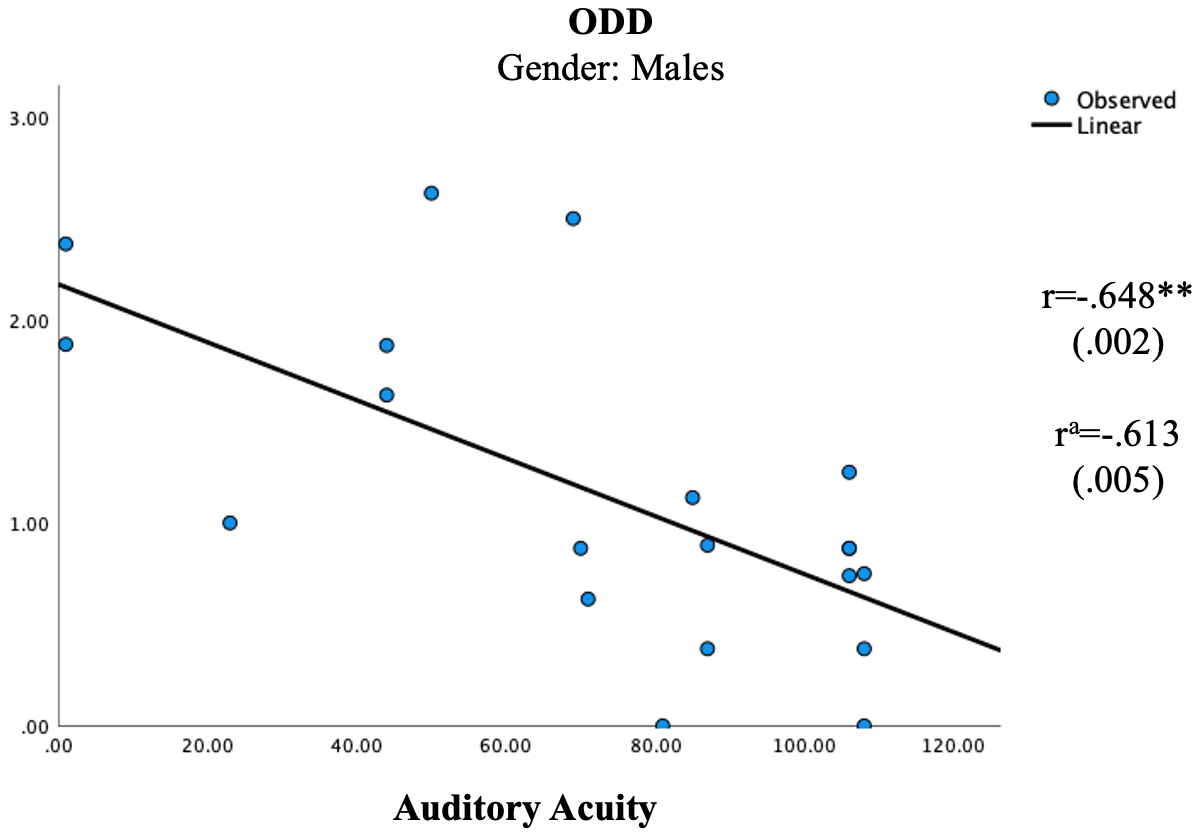
Significant correlations in unisensory processing in the male group. ^a^Using age as a control variable. ^**^p < 0.005.

In the female group, the independent unisensory measurements showed two significant correlations with the parental ratings (Table 5; Figures 1, 2) whereas the scales showed no significant correlation (Table 6). A positive correlation was seen between Auditory Elasticity and Social Motivation (r = 0.519, *p* = 0.023) and a negative correlation was seen between Auditory Acuity and Social Communication (r = −0.535, *p* = 0.022) In the male group, no significant correlations were seen with the Social Responsiveness Scale; however, the unisensory processing showed a negative correlation between the specific measurement of Auditory Acuity and ODD (r = −0.613, *p* = 0.005; Table 4) as well as between the auditory Scale of Maintenance and ODD (r = −0.481, *p* = 0.037; Table 5).

**Table 5:** Pearson correlation table between the independent unisensory IVA-measurements and the parental rating scales^a^.

	Females						Males					
	Elasticity		Acuity		Focus		Elasticity		Acuity		Focus	
Variable (*N*)	Auditory (20)	Visual (20)	Auditory (19)	Visual (20)	Auditory (20)	Visual (20)	Auditory (20)	Visual (19)	Auditory (20)	Visual (20)	Auditory (20)	Visual (20)
Social awareness	0.169	0.228	−0.310	0.218	−0.102	−0.055	0.194	0.228	0.156	−0.050	−0.084	0.142
Social communication	0.394	0.349	**−0.535** ^*^(0.022)	0.270	−0.373	−0.003	0.049	0.151	−0.091	−0.374	−0.232	0.234
Social cognition	0.188	0.271	−300	0.194	−0.226	0.034	0.098	0.192	0.115	−0.209	−0.220	0.288
Social motivation	**0.519** ^*^(0.023)	0.434	−0.170	0.387	0.022	0.330	0.276	0.030	−0.009	−0.040	−0.235	0.133
Social rigidity	0.402	0.367	−0.388	0.265	−0.122	0.086	0.234	0.121	0.090	−0.050	−0.337	0.428
Hyperactivity	0.172	0.270	−0.318	0.276	**−0.464** ^*^(0.045)	−0.111	−0.015	−0.050	0.042	0.136	−0.295	0.311
Inattention	0.161	0.333	−0.347	0.213	−0.321	0.006	0.004	0.354	−0.176	−0.025	0.065	0.279
ODD	0.054	**0.460** ^*^(0.047)	−0.236	0.270	−0.168	0.144	−0.452 (0.052)	0.028	**−0.613** ^**^(0.005)	−0.320	−0.076	−0.289

Pearson r displayed in table. Significant correlations are highlighted and includes (*p*-values).

a Using age as a covariable.

* *p* < 0.05.

** *p* < 0.005.

**Table 6:** Correlation table between the complex unisensory IVA Scales and the parental rating scales^a^.

	Females				Males			
	Scale of competence		Scale of maintenance		Scale of competence		Scale of maintenance	
Variable (*N*)	Auditory (20)	Visual (20)	Auditory (19)	Visual (20)	Auditory (20)	Visual (20)	Auditory (20)	Visual (20)
Awareness	0.310	0.040	−0.008	0.064	0.135	−0.050	0.238	0.104
Communication	0.097	0.100	−0.405 (0.095)	0.151	−0.005	0.046	0.093	−0.140
Cognition	0.125	0.204	−0.404 (0.096)	0.112	0.114	0.078	0.149	−0.008
Motivation	0.281	0.288	0.117	0.448 (0.055)	−0.022	0.270	0.055	0.170
Rigidity	0.258	0.247	−0.249	0.170	−0.005	0.302	0.194	0.191
Hyperactivity	0.125	−0.011	−0.239	0.039	−0.114	0.345	0.143	0.206
Inattention	−0.026	0.148	−0.374	0.084	0.082	0.275	−0.099	0.081
ODD	0.177	0.298	−0.308	0.209	−0.261	−0.143	**−0.481** ^*^ **(0.037)**	−0.383

Pearson r displayed in table. Significant correlations are highlighted and includes (*p*-values).

a Using age as a covariable.*p < 0.05.

**Figure 3:**
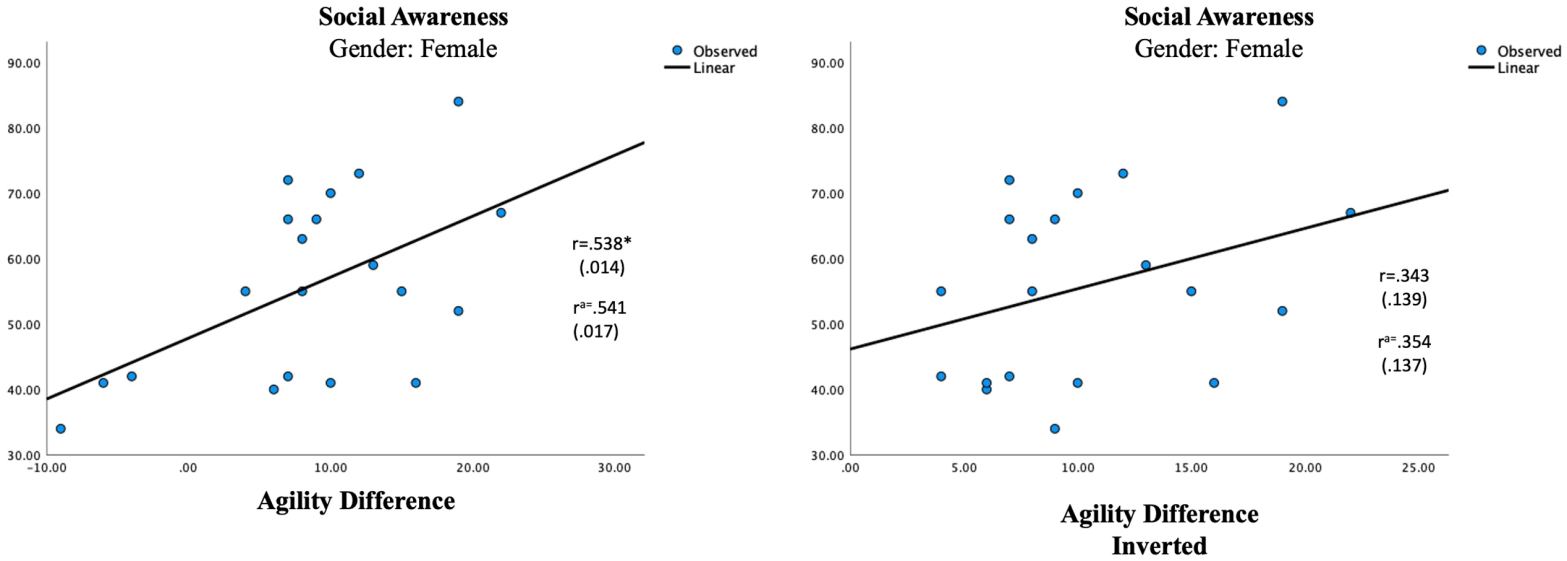
Regression analysis for multisensory processing in the female group. Social awareness and agility different vs. social awareness and agility difference inverted. ^a^Using age as a control variable. ^*^p ≤ 0.05.

**Figure 4:**
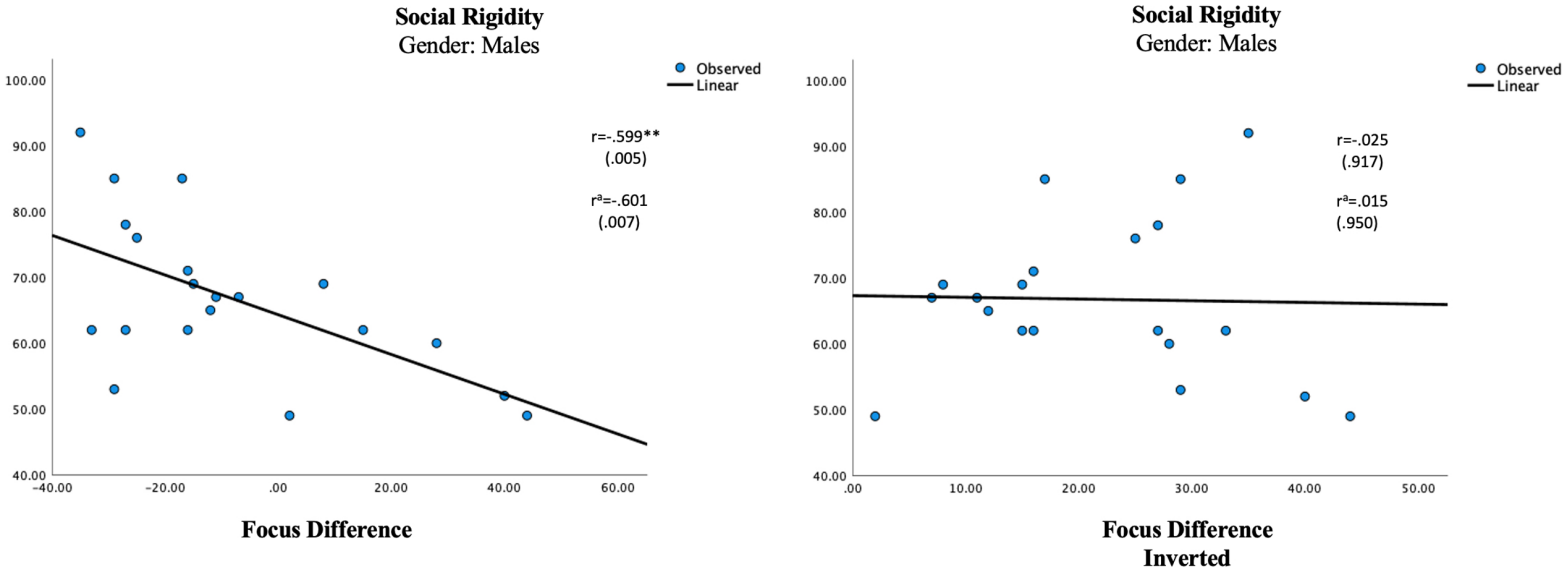
Regression analysis for multisensory processing in males. Social rigidity and focus different score vs. social rigidity and focus difference inverted score. ^a^Using age as a control variable. ^**^p ≤ 0.01.

The difference score, representing the multisensory processing showed one significant correlation in the female group. An auditory dominance in Agility was significantly associated with Social Awareness in the female group (r = 0.541, *p* = 0.017; Table 7; Figure 3). In the male group, a visual dominance in Focus was significantly associated with Social Rigidity (r = −0.601, *p* = 0.007; Table 7; Figure 4).

**Table 7:** Correlation table between the multisensory IVA-measurements, auditory-visual (A-V) difference score and the parental scales^a^.

	Females				Males			
	Focus A-V Difference score		Agility A-V Difference score		Focus A-V Difference score		Agility A-V Difference score	
Variable (*N*)	Biased (20)	Unbiased (20)	Biased (20)	Unbiased (20)	Biased (20)	Unbiased (20)	Biased (20)	Unbiased (20)
Awareness	−0.044	−0.212	**0.541** ^*^ **(0.017)**	0.354	−0.180	−0.052	−0.074	0.022
Communication	−0.392 (0.097)	0.207	0.217	0.016	−0.362	0.073	0.119	0.061
Cognition	−0.263	0.244	0.201	0.000	−0.400	−0.066	−0.039	−0.105
Motivation	−0.323	0.097	0.117	−0.218	−0.277	0.202	−0.448 (0.054)	−0.291
Rigidity	−0.255	0.170	0.162	−0.008	**−0.601** ^**^ **(0.007)**	0.015	−0.349	−0.288
Hyperactivity	−0.358	0.117	0.202	0.142	**−0.471** ^*^ **(0.042)**	0.000	−0.199	−0.165
Inattention	−0.400 (−090)	0.288	−0.256	−0.231	−0.192	0.215	0.017	0.083
ODD	−0.337	0.282	0.023	−0.179	0.193	**0.459** ^*^ **(0.048)**	0.355	0.260

Pearson r displayed in table. Significant correlations are highlighted and includes (*p*-values).

a Using age as a covariable.*p < 0.05.**p < 0.01.

## Discussion

The results confirm gender differences in unisensory vs. multisensory processing with females showing a superior auditory processing associated with better social communication skills in unisensory processing (Figure 1), whereas the multisensory processing was associated with more problems within social awareness (Figure 3). In the male group, visual processing showed no correlation with social skills in the unisensory processing (Figure 2). In the multisensory processing, a visual dominance was associated with difficulties in social rigidity (Figure 4). The lack of gender differences in the rating of Social Responsiveness indicates both groups show the same number of difficulties.

In the female group, a unisensory processing advantage in Auditory Acuity was associated with less rated problems in Social Communication, the males did not show this correlation (Table 5; Figures 1, 2). This is in line with our hypothesis of ASD females showing the same unisensory processing advantage in social communication as is seen in previous studies of TD women (McGivern et al., 2019; Siedlecki et al., 2019; Thornton et al., 2019). Auditory Acuity has previously been associated with language comprehension (Lee et al., 2018; Ayasse et al., 2019) so, a process such as language, heavily relying on auditory information will naturally be favored by those having a superior auditory processing. However, in the male group, a better performance in Auditory Acuity was associated with less rated problems of ODD (r = −0.613, *p* = 0.005; Figure 2), which is in line with our hypothesis of males having a superior visual processing blocking out auditory information. A higher score of auditory acuity in males will be representing a person with a visual dominance of lesser degree, still limiting the auditory information but perhaps reducing autistic behaviors, resulting in less rated problems within ODD. However, the symptoms being rated in Oppositional defiant disorder [American Psychiatric Association (APA), 2013] differs from other psychiatric symptoms since it is a diagnose based on parental interpretation of a child’s intentions and feelings rather than an observation of a specific concrete behavior, hence the measure becomes less specific. Since parental assessments of teen aged children have shown to be influenced by the child’s gender due to preconceptions about the underlying cause for the behaviour (Geelhand et al., 2019) we must count that in as a possible explanation for the results. However, the lack of differences in the parental ratings of Social Responsiveness speaks against that theory, in fact, looking at the actual scores, the females were indeed higher rated than the males in both ODD and Social Communication (Table 3).

In the female group, two significant correlations of the opposite direction were also found in the unisensory processing. A higher performance in Auditory and Visual Elasticity was associated with higher ratings of problems within Social Motivation (r = 0.519, *p* = 0.023) and ODD, respectively (r = 0.460, *p* = 0.047). Elasticity has previously been associated with the use of “Camouflaging strategies” (Hull et al., 2021). Since camouflaging strategies are known to cause exhaustion, stress, and anxiety (Rynkiewicz et al., 2016) it seems reasonable to assume that a higher performance in Auditory Elasticity would be associated with higher ratings of problems within Social Motivation. The association between a *higher* performance in Visual Elasticity and ODD seen in the female group stands in contrast to the male on-the-verge of being significant association between a *lower* performance in *Auditory* Elasticity and higher rated problems within ODD (r = −0.452, *p* = 0.052). Again, the lack of gender differences in the parental ratings makes us turn to other explanations than parental preconceptions of their children for interpretations. Difficulties within language and flexibility have previously been identified as risk factors impacting ODD in TD boys, whereas only flexibility was identified as a risk factor in TD girls (Kerekes et al., 2014). As elasticity is a measure of flexibility and auditory acuity is associated with speech comprehension (Lee et al., 2018; Ayasse et al., 2019), our results are in line with previous research as far as the male correlations go. However, in the female group, a higher performance in visual flexibility is associated with ODD rather than a lower performance. How can this be explained? A farfetched but still reasonable interpretation can perhaps be found by applying the “extreme female brain” (Floris et al., 2018) vs. “extreme male brain” theories (Shams et al., 2000; Alais and Burr, 2004). By looking at the two groups’ overall performance in Elasticity, the male performance in auditory and visual elasticity are equally good, whereas in the female group, there is an auditory superiority (Figure 3). The female correlation between a higher Visual Elasticity and ODD symptoms could then be a result caused by those females showing more of a male structured brain; hence, the more “male-like” a female autistic child’s brain is, the more similar will the outcome of behavior also be.

To get a more complex measure of unisensory processing, we included measures of performance in scales representing low- and high-demanding unisensory processing, respectively. Although no significant correlations were seen in the female group (Table 6) they outperformed the male group in Scale of Maintenance having a 20-point higher average score (Table 3). The female group showed a couple of non-significant correlations with Auditory and Visual Maintenance [Social communication (r = −0.405) and Social Motivation (r = 0.448), respectively] mirroring those seen in the correlations with the specific measurements of Auditory Acuity and Visual Elasticity (Table 6). In the male group, a significant negative correlation was seen between auditory Scale of Maintenance, representing low-demanding tasks and ODD (r = −0.481, *p* = 0.037; Table 6). Since both groups show correlations that are smaller and less significant than the specific measurements of Acuity and Elasticity, we can assume that the specific IVA measurements are of more value in predicting psychiatric symptoms than the scales built up by several aspects of auditory vs. visual performance. This is noteworthy for future research since it implies it is the superiority of specific auditory vs. visual measurements that are of interest in the association with social responsiveness rather than an overall auditory or visual performance. However, an interesting observation in the female group are the two similar non-significant correlations between Auditory Scale of Maintenance and Social Cognition (r = −0.404, *p* = 0.096), respectively, and Social Communication (r = −0.405, *p* = 0.095). In the correlations with the specific measurements, Social Communication showed a significant correlation with Auditory Acuity (r = −0.535, *p* = 0.22) whereas Social Cognition showed a much smaller non-significant correlation (r = −0.300) which indicates Social Cognition might be better predicted with a more complex measure of auditory performance.

Moving over to the results seen in multisensory processing, we find support for the belief of an extreme female vs. male brain. The multisensory processing represents situations where both auditory and visual information need to be processed. In the female group a superior auditory Agility was associated with more rated problems within Social Awareness, whereas in the male group a superior visual Focus was associated with more rated problems within Social Rigidity (Table 7). This supports our belief that ASD is portrayed through different gender phenotypes. It is also in line with previous research of gender differences in ASD showing males to have more rigid behaviors than females (Werling and Geschwind, 2013; Lockwood Estrin et al., 2021). However, the parental ratings show no significant difference between the groups regarding social rigidity, indicating the females show as many rigid behaviors as males (Table 3). A reasonable explanation can be related to gender differences in the portrayal of rigidity. Previous research of children with ASD has noted gender differences in the portrayal of rigid behaviors where boys are more inclined to show stereotyped behaviors and restricted interests, whereas girls are more compulsive insisting on sameness and having self-injurious behavior (Antezana et al., 2019). Since the phenotypes of female rigidity are not as well represented in rating scales as male rigidity is, it makes sense other scales might be more suitable for rating females. An auditory dominance in the more complex multisensory measure of Agility might then indeed be associated with higher-rated problems within Social Awareness in only ASD females, whereas in males a visual dominance in Focus is related to higher-rated problems in Social Rigidity. The fact that we see gender differences in the correlations between cognitive abilities and social difficulties gives further support to our believe that ASD is differently portrayed in females than in males and that different cognitive profiles produce different kinds of problems.

To be sure our results represent a modality dominance rather than any modality imbalance, we inversed all the negative difference scores to produce an unbalanced measure (Table 7) which were then correlated with the parental ratings. In the female group, the inverted measurement of Agility showed a smaller, non-significant correlation (r = 0.354, *p* = 0.137) with Social Awareness and in the male group, the new measure showed no correlation at all (r = 0.015). The new lower association seen in the female group is in line with research of language processing claiming females use bilateral processing in language processing, relying on both auditory and visual information to a higher degree than males (Burman et al., 2008; Koles et al., 2010; Ross et al., 2015). In our hypothesis, we suggested females need information from both modalities when processing complex settings and if that is true any auditory–visual imbalance in multisensory settings should produce some difficulties which is also what the results show. The lack of correlation between the inverted score and social rigidity in the male group signifies that it is mostly a visual dominance that will affect the language process in males which again is in line with the research mentioned above, claiming males to be more reliant on auditory information in the process of language (Burman et al., 2008; Koles et al., 2010; Ross et al., 2015).

In the female group, a couple of non-significant trends were also seen between a visual dominance in Focus and difficulties with Social Communication and Inattention. Since the correlations are seen in the measurement of Focus, just as in the male group, again one can speculate if those are represented by females being more of the ASD male phenotype.

The result of our study supports the belief of a female phenotype of autism portrayed in form of an extreme female brain making it easier to process unisensory information in case of a sensory disturbance. This could very well be considered as females being biologically protected from ASD as some claim them to be (Baron-Cohen et al., 2011; Werling and Geschwind, 2013; Werling, 2016); however, one would think that a protection would mean females also have less rated problems within social responsiveness which is not the case. The question we therefor need to ask is whether a unisensory processing advantage is in fact an advantage in a modern complex society of today? As it seems, an auditory–visual processing *advantage* may very well be the downfall in an assessment procedure, allowing female patients to pass at subclinical levels, despite showing an equal or sometimes even higher degree of difficulties than ASD males (Hanley, 2016; Beggiato et al., 2017; Lai and Szatmari, 2020).

Our study showed only one significant gender difference regarding the IVA-performance (Scale of Maintenance) even if the females produced higher scores in all but one measurement (Visual Elasticity). A higher power might have provided more significant differences; however, it is worth noting that most of the IVA-performances in the female group are still well below the average performance of a norm curve (Figure 1). This is important to acknowledge when discussing if females are underrepresented in ASD or not. Females might not be affected in the same way as males, but it does not mean that they are not affected at all.

The thought of females being protected in less complex social settings, still having difficulties in more complex settings raises several questions. For example, what does the concept of “being protected” include and how do you know when that concept is fulfilled? What does it imply to be able to handle a less complex social setting? Does it mean someone is able to handle a social setting in the same way as a TD person? Or does it mean that someone can adapt in a way that from the outside is perceived as a TD behavior but lacking the ability to account for their own feelings and thoughts, leaving them exhausted and vulnerable to develop psychiatric problems? Previous research of Camouflaging behavior in ASD is supportive of the latter definition rather than the first. Camouflaging strategies, the ability to behave in a seemingly flexible way, are said to blur social difficulties and inadvertently contribute to misconstruing important clinical and eligibility decisions (Hull et al., 2017; Young et al., 2018). The lack of flexibility is said to make the individual exhausted and in risk of developing various depression- and anxiety-related disorders (Rynkiewicz et al., 2019).

In the theory of social motivation, they differentiate between social settings requiring unisensory vs. and multisensory processing and refer to less complex social behavior and more complex social behavior, respectively (Tamir and Hughes, 2018). While less complex social behavior is referred to situations that do not require different perspectives, complex social behavior reflects situations where a person needs to take more perspectives into account before action is taken and therefore requires the integration of information from different modalities (Tamir and Hughes, 2018).

A less complex social setting might then be represented by a situation where the individual can understand what is expected here and now and behave in such a way. In the short run, it is not a problem since everybody can handle to set their own emotions aside occasionally, but in the long run, it will create a problem. For several “less complex social settings” to make sense, the individual must see to the overall perspective and be able to account for their own feelings and thoughts. A more complex setting may then be built up by several less complex settings. In order to uphold a psychiatric wellbeing, the individual need to be able to grasp the overall perspective and understand how to adapt to the more complex social setting of which the “less complex setting” is a part.

The ASD assessment procedure of today has been critiqued by those meaning that it is predominantly focused on male ASD symptoms. This has raised the request for more research of how ASD is portrayed in females (Moseley et al., 2018; Young et al., 2018).

Perhaps another important question to be raised is whether the assessment procedure of today can provide such a complex social setting that might be needed for a female ASD patient to be detected? The Autism Diagnostic Observation Schedule (ADOS-2) used in ASD assessment of today includes a certain number of tasks/questions for the patient to adhere to while the assessor assesses whether the patient is showing proof of autistic behavior or not (Lord et al., 2012). Since an ADOS-2 observation only involves the patient and the assessor, takes about 1 h to implement, and the different tasks are in no way connected to each other, it shows every sign of being a representative of “less complex social settings.”

By creating an assessment procedure that provides more complex social settings we might be able to detect females at an earlier stage, reducing their risk of them being severely injured. A missed diagnose will not only prolong the course of disease, but it will also put the patient at risk of developing severe psychiatric symptoms (Rynkiewicz et al., 2019; Sveriges Kommuner och Regioner, 2021) putting an extra burden on society since patients with severe psychiatric unhealthy are less likely to uphold a job and more likely to need psychiatric care (Lanctôt et al., 2013; Bailey et al., 2018).

### Conclusion

Our result supports a gender-specific understanding of ASD, suggesting a female auditory processing advantage is of importance in the understanding of why females are considered to be “biologically protected” from ASD. However, the results indicate that the social advantages provided by having an auditory processing advantage are limited to social settings of less complexity. More complex social settings seem to be as difficult to handle for ASD females as they are for ASD males.

The lack of significant group differences regarding social responsiveness indicates both groups show an equal level of social functioning despite the superior auditory processing seen in the female group. We suggest the autism assessment procedures of today need to be reworked to include observations of behavior in settings of higher complexity level to be able to detect the difficulties of patients with a feminine ASD profile. As of today, the female “protective” factor might as well be working as a pitfall for those who “passes” the ASD assessment and are left without a diagnosis.

### Strength and limitations

To avoid the DSM-5 criteria possibly screening out children we aimed to reach out to, our design included all children coming for an ASD assessment rather than children already diagnosed. We might still have missed several children since the triaging process also is depending on the DSM-5 criteria of ASD. Another strength of this study is the use of the computerized IVA-test. The children have been exposed to the exact same testing procedure which reduces the risk of the assessor affecting the results. The parental scales are used in the same way as they are used in an ASD assessment, hence eventual problems related to parental differences in rating will mirror the reality of using parental assessments as a diagnostic tool.

This study also has limitations, one being the size of the study. With the possible lack of statistical power in this study, it cannot be excluded that statistical correlations, now displaying trends toward a correlation in some cases, may have provided more clearly, significant results in a larger study sample. Also, in a small study, covariables such as age are difficult to control for. Even though we used age as a control variable in all correlations, the possibility of odd cases skewing the results is much higher in a small population than in a larger size population. We tried to secure this by excluding a couple of IVA-measures that were outliers. The high number of subjects (25%) being excluded from the study due to invalid IVA-results or an inability to go through with the IVA-test is also a matter of concern. Hypothetically these subjects can be sharing a specific autistic trait causing difficulties to succeed with an IVA-test; hence, when excluding a group of subjects with similar behavior, we run the risk of getting skewed results. However, there were about the same number of boys and girls that were excluded, indicating that the same difficulties are present in both genders. The lack of a representation of a specific autistic trait will therefore be seen in both groups. Further, the lack of a measure of the child’s socioeconomic status could possibly have helped us understand the parental ratings to a better degree perhaps giving us a better understanding of parental abilities to support their child. We tried to reduce the effect of selection bias by using children from the same socioeconomic area, with an IQ of 85 and above, as well as making it easy to participate, no extra travels were needed. A final limitation worth noting is the IQ-level used as an exclusion criterion. The result of the study can only be related to people with an IQ-level within the normal variation or above.

We believe our study has contributed with a different gender perspective of ASD, showing results that are in line with previous research and have the possibility to add a great deal of understanding of female ASD. Future research should focus on ASD gender differences in audio-visual language processing in social settings of different complexity. Also, there is a need for future research to focus on the ASD assessment procedures which is in need for a radical update to be able to pick up those children that today go undetected.

## Data availability statement

The raw data supporting the conclusions of this article will be made available by the authors, without undue reservation.

## Ethics statement

The studies involving human participants were reviewed and approved by the Regional Ethics Committee in Lund. Written informed consent to participate in this study was provided by the participants’ legal guardian/next of kin.

## Author contributions

SÅ has designed, collected, and analyzed the material and also written the article. AH and EC-K have been supervising the project, as well as proof read the article. All authors contributed to the article and approved the submitted version.

## Funding

AH has a position as a researcher at Lund University which has been sponsored by AB Svenska Spel, the state-owned gambling company of Sweden, and AH also has funding from the research council from AB Svenska Spel and from the research council of Systembolaget AB, the alcohol monopoly of Sweden. All authors have funding from the research council of Svenska Spel. The study was enabled by funding from FOU Region Skåne and Thure Carlsson foundation.

## Conflict of interest

The authors declare that the research was conducted in the absence of any commercial or financial relationships that could be construed as a potential conflict of interest.

## Publisher’s note

All claims expressed in this article are solely those of the authors and do not necessarily represent those of their affiliated organizations, or those of the publisher, the editors and the reviewers. Any product that may be evaluated in this article, or claim that may be made by its manufacturer, is not guaranteed or endorsed by the publisher.

## References

[ref1] Alais D. Burr D. (2004). The ventriloquist effect results from near-optimal bimodal integration. Curr. Biol. 14, 257–262. doi: 10.1016/j.cub.2004.01.029, PMID: 14761661

[ref2] American Psychiatric Association (APA) (2013). DSM-5. Diagnostic and statistical manual of mental disorders (5th ed.). Arlington, VA: American Psychiatric Association.

[ref3] Antezana L. Factor R. S. Condy E. E. Strege M. V. Scarpa A. Richey J. A. (2019). Gender differences in restricted and repetitive behaviors and interests in youth with autism. Autism Res. 12, 274–283. doi: 10.1002/aur.2049, PMID: 30561911

[ref4] Antshel K. M. Russo N. (2019). Autism Spectrum disorders and ADHD: overlapping phenomenology, diagnostic issues, and treatment considerations. Curr. Psychiatry Rep. 21:34. doi: 10.1007/s11920-019-1020-5, PMID: 30903299

[ref5] Asperger H. Frith U. T. (1991). “Autistic psychopathy’ in childhood” in Autism and Asperger syndrome. ed. Frith U. (New York, NY: Cambridge University Press), 37–92.

[ref6] Ayasse N. Penn L. Wingfield A. (2019). Variations within Normal hearing acuity and speech comprehension: an exploratory study. Am. J. Audiol. 28, 369–375. doi: 10.1044/2019_AJA-18-0173, PMID: 31091111 PMC6802869

[ref7] Ayres A. J. (1979). Sensory integration and the child. Western Psychological Services. Los Angeles.

[ref8] Bailey R. Sharpe D. Kwiatkowski T. Watson S. Dexter Samuels A. Hall J. (2018). Mental health care disparities now and in the future. J. Racial Ethn. Health Disparities 5, 351–356. doi: 10.1007/s40615-017-0377-6, PMID: 28634875

[ref9] Balasco L. Provenzano G. Bozzi Y. (2020). Sensory abnormalities in autism Spectrum disorders: a focus on the tactile domain, from genetic mouse models to the clinic. Front. Psych. 10:1016. doi: 10.3389/fpsyt.2019.01016, PMID: 32047448 PMC6997554

[ref10] Baron-Cohen S. (2003). The essential difference: Men, women and the extreme male brain (Penguin, London).

[ref11] Baron-Cohen S. Lombardo M. V. Auyeung B. Ashwin E. Chakrabarti B. Knickmeyer R. (2011). Why are autism spectrum conditions more prevalent in males? Biology 9:e1001081. doi: 10.1371/journal.pbio.1001081, PMID: 21695109 PMC3114757

[ref12] Bebko J. M. Weiss J. A. Demark J. L. Gomez P. (2006). Discrimination of temporal synchrony in intermodal events by children with autism and children with developmental disabilities without autism. J. Child Psychol. Psychiatry 47, 88–98. doi: 10.1111/j.1469-7610.2005.01443.x, PMID: 16405645

[ref13] Beggiato A. Peyre H. Maruani A. Scheid I. Rastam M. Amsellem F.. (2017). Gender differences in autism spectrum disorders: divergence among specific core symptoms. Autism Res. 10, 680–689. doi: 10.1002/aur.1715, PMID: 27809408

[ref14] Boyle M. H. Cunningham C. E. Georgiades K. Cullen J. Racine Y. Pettingill P. (2009). The brief child and family phone interview (BCFPI): 2. Usefulness in screening for child and adolescent psychopatholog. J. Child Psychol. Psychiatry 50, 424–431. doi: 10.1111/j.1469-7610.2008.01971.x, PMID: 19175807

[ref15] Burman D. D. Bitan T. Booth J. R. (2008). Sex differences in neural processing of language among children. Neuropsychologia 46, 1349–1362. doi: 10.1016/j.neuropsychologia.2007.12.021, PMID: 18262207 PMC2478638

[ref16] Chiarotti F. Venerosi A. (2020). Epidemiology of autism Spectrum disorders: a review of worldwide prevalence estimates since 2014. Brain Sci. 10:274. doi: 10.3390/brainsci10050274, PMID: 32370097 PMC7288022

[ref17] Cola M. L. Plate S. Yankowitz L. Petrulla V. Bateman L. Zampella C. J.. (2020). Sex differences in the first impressions made by girls and boys with autism. Mol. Autism. 11:49. doi: 10.1186/s13229-020-00336-3, PMID: 32546266 PMC7298946

[ref18] Constantino J. N. Davis S. Todd R. Schindler M. Gross M. Brophy S.. (2003). Validation of a brief quantitative measure of autistic traits: comparison of the social responsiveness scale with the autism diagnostic interview-revised. J. Autism Dev. Disord. 33, 427–433. doi: 10.1023/A:1025014929212, PMID: 12959421

[ref19] Constantino J. N. Gruber C. P. (2012). Social responsiveness scale: (SRS)-2, Torrance, CA: Western Psychological Services. 106.

[ref20] Cunningham C. E. Boyle M. H. Hong S. Pettingill P. Bohaychuk D. (2009). The brief child and family phone interview (BCFPI): 1. Rationale, development, and description of a computerized children's mental health intake and outcome assessment tool. J. Child Psychol. Psychiatry 50, 416–423. doi: 10.1111/j.1469-7610.2008.01970.x, PMID: 19017368

[ref21] De Boer-Schellekens L. Keetels M. Eussen M. Vroomen J. (2013). No evidence for impaired multisensory integration of low level audiovisual stimuli in adolecsents and young adults with autism spectrum disorders. Neuropsychologia 51, 3004–3013. doi: 10.1016/j.neuropsychologia.2013.10.005, PMID: 24157536

[ref22] De Niear M. A. Gupta P. B. Baum S. H. Wallace M. T. (2018). Perceptual training enhances temporal acuity for multisensory speech. Neurobiol. Learn. Mem. 147, 9–17. doi: 10.1016/j.nlm.2017.10.016, PMID: 29107704

[ref23] Dean M. Harwood R. Kasari C. (2017). The art of camouflage: gender differences in the social behaviors of girls and boys with autism spectrum disorder. Autism 21, 678–689. doi: 10.1177/1362361316671845, PMID: 27899709

[ref24] DiCriscio A. S. Troiani V. (2017). Pupil adaptation corresponds to quantitative measures of autism traits in children. Sci. Rep. 7:6476. doi: 10.1038/s41598-017-06829-1, PMID: 28743966 PMC5526922

[ref25] Dunn W. (2001). The sensations of everyday life: empirical, theoretical, and pragmatic considerations. Am. J. Occup. Ther. 55, 608–620. doi: 10.5014/ajot.55.6.608, PMID: 12959225

[ref26] Falter C. M. Elliott M. A. Bailey A. J. (2012). Enhanced visual temporal resolution in autism Spectrum disorders. PLoS One 7:e32774. doi: 10.1371/journal.pone.0032774, PMID: 22470425 PMC3309999

[ref27] Feldman J. I. Dunham K. Cassidy M. Wallace M. T. Liu Y. Woynaroski T. G. (2018). Audiovisual multisensory integration in individuals with autism spectrum disorder: a systematic review and meta-analysis. Neurosci. Biobehav. Rev. 95, 220–234. doi: 10.1016/j.neubiorev.2018.09.020, PMID: 30287245 PMC6291229

[ref28] Floris D. L. Lai M. C. Nath T. (2018). Network-specific sex differentiation of intrinsic brain function in males with autism. Mol. Autism. 9:17. doi: 10.1186/s13229-018-0192-x, PMID: 29541439 PMC5840786

[ref29] Foss-Feig J. H. Schauder K. B. Key A. P. Wallace M. T. Stone W. L. (2017). Audition-specific temporal processing deficits associated with language function in children with autism spectrum disorder. Autism Res. 10, 1845–1856. doi: 10.1002/aur.1820, PMID: 28632303 PMC6007978

[ref30] Geelhand P. Bernard P. Klein O. van Tiel B. Kissine M. (2019). The role of gender in the perception of autism symptom severity and future behavioral development. Mol. Autism. 10:16. doi: 10.1186/s13229-019-0266-4, PMID: 30976383 PMC6439965

[ref31] Greenberg D. M. Warrier V. Allison C. Baron-Cohen S. (2018). Testing the empathizing-systemizing theory of sex differences and the extreme male brain theory of autism in half a million people. Proc. Natl. Acad. Sci. U. S. A. 115, 12152–12157. doi: 10.1073/pnas.1811032115, PMID: 30420503 PMC6275492

[ref32] Hall C. L. Guo B. Valentine A. Z. Groom M. J. Daley D. Sayal K.. (2020). The validity of the SNAP-IV in children displaying ADHD symptoms. Assessment 27, 1258–1271. doi: 10.1177/1073191119842255, PMID: 30991820

[ref33] Hanley J. L. (2016). Autism, females, and the DSM-5: gender bias in autism diagnosis. Soc. Work. Ment. Health 14, 396–407. doi: 10.1080/15332985.2015.1031858

[ref34] Hickok G. Poeppel D. (2007). The cortical organization of speech processing. Nat. Rev. Neurosci. 8, 393–402. doi: 10.1038/nrn2113 17431404

[ref35] Hull L. Petrides K. V. Allison C. Smith P. Baron-Cohen S. Lai M.-C.. (2017). “Putting on my best normal”: social camouflaging in adults with autism spectrum conditions. J. Autism Child. Schizophr. 47, 2519–2534. doi: 10.1007/s10803-017-3166-5, PMID: 28527095 PMC5509825

[ref36] Hull L. Petrides K. V. Mandy W. (2021). Cognitive predictors of self-reported camouflaging in autistic adolescents. Autism Res. 14, 523–532. doi: 10.1002/aur.2407, PMID: 33047869

[ref37] IBM Corp. (2019). IBM SPSS statistics for Macintosh, Version 26.0. Armonk, NY: IBM Corp.

[ref38] Jain C. Priya M. B. Joshi K. (2020). Relationship between temporal processing and phonological awareness in children with speech sound disorders. Clin. Linguist. Phon. 34, 566–575. doi: 10.1080/02699206.2019.1671902, PMID: 31566027

[ref39] Jolliffe T. Baron-Cohen S. J. (1997). Are people with autism and Asperger syndrome faster than normal on the embedded figures test? J. Child Psychol. Psychiatry 38, 527–534. doi: 10.1111/j.1469-7610.1997.tb01539.x, PMID: 9255696

[ref40] Joseph R. M. Keehn B. Connolly C. Wolfe J. M. Horowitz T. S. (2009). Why is visual search superior in autism spectrum disorder? Dev. Sci. 12, 1083–1096. doi: 10.1111/j.1467-7687.2009.00855.x, PMID: 19840062 PMC12049234

[ref41] Kaldy Z. Giserman I. Carter A. S. Blaser E. (2016). The mechanisms underlying the ASD advantage in visual search. J. Autism Dev. Disord. 46, 1513–1527. doi: 10.1007/s10803-013-1957-x, PMID: 24091470 PMC3976471

[ref42] Kerekes N. Lundström S. Chang Z. Tajnia A. Jern P. Lichtenstein P.. (2014). Oppositional defiant-and conduct disorder-like problems: neurodevelopmental predictors and genetic background in boys and girls, in a nationwide twin study. PeerJ 2:e359. doi: 10.7717/peerj.359, PMID: 24795851 PMC4006222

[ref43] King A. J. Hammond-Kenny A. Nodal F. R. (2019). “Multisensory processing in the auditory cortex” in Multisensory processes: The auditory perspective. eds. Lee A. Wallace M. Coffin A. Popper A. Fay R., vol. 68 (Cham: Springer)

[ref44] Koles Z. J. Lind J. C. Flor-Henry P. (2010). Gender differences in brain functional organization during verbal and spatial cognitive challenges. Brain Topogr. 23, 199–204. doi: 10.1007/s10548-009-0119-0, PMID: 19943102

[ref45] Kwakye L. D. Foss-Feig J. H. Cascio C. J. Stone W. L. Wallace M. T. (2011). Altered auditory and multisensory temporal processing in autism spectrum disorders. Front. Integr. Neurosci. 4:129. doi: 10.3389/fnint.2010.00129 21258617 PMC3024004

[ref46] Lai M. C. Lombardo M. V. Ruigrok A. N. Chakrabarti B. Auyeung B. Szatmari P.. (2017). Quantifying and exploring camouflaging in men and women with autism. Autism 21, 690–702. doi: 10.1177/1362361316671012, PMID: 27899710 PMC5536256

[ref47] Lai M. C. Lombardo M. V. Suckling J. Ruigrok A. N. Chakrabarti B. Ecker C.. (2013). Biological sex affects the neurobiology of autism. Brain 136, 2799–2815. doi: 10.1093/brain/awt216, PMID: 23935125 PMC3754459

[ref48] Lai M. C. Szatmari P. (2020). Sex and gender impacts on the behavioral presentation and recognition of autism. Curr. Opin. Psychiatry 33, 117–123. doi: 10.1097/YCO.0000000000000575, PMID: 31815760

[ref49] Lanctôt N. Bergeron-Brossard P. Sanquirgo N. Corbière M. (2013). Causal attributions of job loss among people with psychiatric disabilities. Psychiatr. Rehabil. J. 36, 146–152. doi: 10.1037/prj0000002, PMID: 23815175

[ref50] Lee Y. S. Wingfield A. Min N. E. Kotloff E. Grossman M. Peelle J. E. (2018). Differences in hearing acuity among "Normal-hearing" Young adults modulate the neural basis for speech comprehension. eNeuro. 5:ENEURO.0263-17.2018. doi: 10.1523/ENEURO.0263-17.2018, PMID: 29911176 PMC6001266

[ref51] Lin Y. Ding H. Zhang Y. (2021). Unisensory and multisensory Stroop effects modulate gender differences in verbal and nonverbal emotion perception. J. Speech Lang. Hear. Res. 64, 4439–4457. doi: 10.1044/2021_JSLHR-20-00338, PMID: 34469179

[ref52] Lockwood Estrin G. Milner V. Spain D. Happé F. Colvert E. (2021). Barriers to autism Spectrum disorder diagnosis for Young women and girls: a systematic review. Rev. J. Autism Dev. Disord. 8, 454–470. doi: 10.1007/s40489-020-00225-8, PMID: 34868805 PMC8604819

[ref53] Loomes R. Hull L. Mandy W. P. L. (2017). What is the male-to-female ratio in autism Spectrum disorder? A systematic review and meta-analysis. J. Am. Acad. Child Psychiatry 56, 466–474. doi: 10.1016/j.jaac.2017.03.013, PMID: 28545751

[ref54] Lord C. Rutter M. DiLavore P. C. Risi S. Gotham K. Bishop S. (2012). Autism diagnostic observation schedule (3rd ed.). Torrance, CA: Western Psychological Services.

[ref55] Marco E. J. Hinkley L. B. Hill S. S. Nagarajan S. S. (2011). Sensory processing in autism: a review of neurophysiologic findings. Pediatr. Res. 69, 48R–54R. doi: 10.1203/PDR.0b013e3182130c54, PMID: 21289533 PMC3086654

[ref56] McDonnell C. G. DeLucia E. A. Hayden E. P. Penner M. Curcin K. Anagnostou E.. (2021). Sex differences in age of diagnosis and first concern among children with autism Spectrum disorder. J. Clin. Child Psychol. 50, 645–655. doi: 10.1080/15374416.2020.1823850, PMID: 33136459

[ref57] McGivern R. F. Mosso M. Freudenberg A. Handa R. J. (2019). Sex related biases for attending to object color versus object position are reflected in reaction time and accuracy. Public Libr. Sci. 14:e0210272. doi: 10.1371/journal.pone.0210272, PMID: 30625223 PMC6326485

[ref58] Meilleur A. Foster N. E. V. Coll S.-M. Brambati S. M. Hyde K. L. (2020). Unisensory and multisensory temporal processing in autism and dyslexia: a systematic review and meta-analysis. Neurosci. Biobehav. Rev. 116, 44–63. doi: 10.1016/j.neubiorev.2020.06.013, PMID: 32544540

[ref59] Moseley R. L. Hitchiner R. Kirkby J. A. (2018). Self-reported sex differences in high-functioning adults with autism: a meta-analysis. Mol. Autism. 9:33. doi: 10.1186/s13229-018-0216-6, PMID: 29796237 PMC5960195

[ref60] Noel J.-P. Stevenson R. Wallace M. (2018). Atypical audiovisual temporal function in autism and schizophrenia: similar phenotype, different cause. Eur. J. Neurosci. 47, 1230–1241. doi: 10.1111/ejn.13911, PMID: 29575155 PMC5980744

[ref61] Ocak E. Eshraghi R. S. Danesh A. Mittal R. Eshraghi A. A. (2018). Central auditory processing disorders in individuals with autism Spectrum disorders. Balkan Med. J. 35, 367–372. doi: 10.4274/balkanmedj.2018.0853, PMID: 29952312 PMC6158468

[ref62] Parish-Morris J. Liberman M. Y. Cieri C. Herrington J. D. Yerys B. E. Bateman L.. (2017). Linguistic camouflage in girls with autism spectrum disorder. Mol. Autism. 8:48. doi: 10.1186/s13229-017-0164-6, PMID: 29021889 PMC5622482

[ref63] Posar A. Visconti P. (2018). Sensory abnormalities in children with autism spectrum disorder. J. Pediatr. 94, 342–350. doi: 10.1016/j.jped.2017.08.008 29112858

[ref64] Robertson C. E. Baron-Cohen S. (2017). Sensory perception in autism. Nat. Rev. Neurosci. 18, 671–684. doi: 10.1038/nrn.2017.112 28951611

[ref65] Ross L. A. Del Bene V. A. Molholm S. Frey H. P. Foxe J. J. (2015). Sex differences in multisensory speech processing in both typically developing children and those on the autism spectrum. Front. Neurosci. 9:185. doi: 10.3389/fnins.2015.00185, PMID: 26074757 PMC4445312

[ref66] Rynkiewicz A. Janas-Kozik M. Słopień A. (2019). Girls and women with autism. Psychiatr. Pol. 53, 737–752. doi: 10.12740/PP/OnlineFirst/95098 31760407

[ref67] Rynkiewicz A. Schuller B. Marchi E. Piana S. Camurri A. Lassalle A.. (2016). An investigation of the ‘female camouflage effect’ in autism using a computerized ADOS-2 and a test of sex/gender differences. Mol. Autism. 7:10. doi: 10.1186/s13229-016-0073-0, PMID: 26798446 PMC4721191

[ref68] Saito M. Hirota T. Sakamoto Y. Adachi M. Takahashi M. Osato-Kaneda A.. (2020). Prevalence and cumulative incidence of autism spectrum disorders and the patterns of co-occurring neurodevelopmental disorders in a total population sample of 5-year-old children. Mol. Autism. 11:35. doi: 10.1186/s13229-020-00342-5, PMID: 32410700 PMC7227343

[ref69] Sandford J. A. Turner A. (2000). Integrated visual and auditory continuous performance test manual. Richmond, VA: Braintrain Inc.

[ref70] Sato M. (2020). The neurobiology of sex differences during language processing in healthy adults: a systematic review and a meta-analysis. Neuropsychologia 140, 107404–107408. doi: 10.1016/j.neuropsychologia.2020.107404 32087207

[ref71] Shams L. Kamitani Y. Shimojo S. (2000). Illusions. What you see is what you hear. Nature 408:788. doi: 10.1038/35048669 11130706

[ref72] Siedlecki K. L. Falzarano F. Salthouse T. A. (2019). Examining gender differences in neurocognitive functioning across adulthood. J. Int. Neuropsychol. Soc. 25, 1051–1060. doi: 10.1017/S1355617719000821, PMID: 31378214 PMC7331091

[ref73] Statistiska Centralbyrån. (2021). Basingstoke Nature Publishing Group. Available at: https://kommunsiffror.scb.se

[ref74] Stevenson R. A. Wallace M. T. Altieri N. (2014). The interaction between stimulus factors and cognitive factors during multisensory integration of audiovisual speech. Front. Psychol. 5:352. doi: 10.3389/fpsyg.2014.00352, PMID: 24817856 PMC4013471

[ref75] Sveriges Kommuner och Regioner. (2021). Ny rapport om skador inom psykiatrisk vård. Available at: https://skr.se/skr/halsasjukvard/patientsakerhet/nyhetsarkivpatientsakerhet/arkivpatientsakerhet/nyrapportomskadorinompsykiatriskvard.50245.html

[ref76] Swanson J. M. Schuck S. Porter M. M. Carlson C. Hartman C. A. Sergeant J. A.. (2012). Categorical and dimensional definitions and evaluations of symptoms of ADHD: history of the SNAP and the SWAN rating scales. Int. J. Educ. Psychol. Assess. 10, 51–70. 26504617 PMC4618695

[ref77] Tamir D. I. Hughes B. L. (2018). Social rewards: from basic social building blocks to complex social behavior. Perspect. Psychol. Sci. 13, 700–717. doi: 10.1177/1745691618776263, PMID: 30415630

[ref78] Tanigawa J. Kagitani-Shimono K. Matsuzaki J. Ogawa R. Ozono K. (2018). Atypical auditory language processing in adolescents with autism spectrum disorder. Clin. Neurophysiol. 129, 2029–2037. doi: 10.1016/j.clinph.2018.05.014, PMID: 29934264

[ref79] Thornton D. Harkrider A. W. Jenson D. E. Saltuklaroglu T. (2019). Sex differences in early sensorimotor processing for speech discrimination. Sci. Rep. 9:392. doi: 10.1038/s41598-018-36775-5, PMID: 30674942 PMC6344575

[ref80] Wallace M. T. Stevenson R. A. (2014). The construct of the multisensory temporal binding window and its dysregulation in developmental disabilities. Neuropsychologia 64, 105–123. doi: 10.1016/j.neuropsychologia.2014.08.005, PMID: 25128432 PMC4326640

[ref81] Wallentine M. (2020). Chapter 6 – Gender differences in language are small but matters for disorders. Handb. Clin. Neurol. 175, 81–102. doi: 10.1016/B978-0-444-64123-6.00007-2 33008545

[ref82] Wechsler D. (2003). Wechsler intelligence scale for children – Fourth edition (WISC-IV). San Antonio, TX: The Psychological Corporation.

[ref83] Werling D. M. (2016). The role of sex-differential biology in risk for autism spectrum disorder. BSD 7:58. doi: 10.1186/s13293-016-0112-8, PMID: 27891212 PMC5112643

[ref84] Werling D. M. Geschwind D. H. (2013). Sex differences in autism spectrum disorders. Curr. Opin. Neurol. 26, 146–153. doi: 10.1097/WCO.0b013e32835ee548, PMID: 23406909 PMC4164392

[ref85] Woynaroski T. Kwakye L. Foss-Feig H. J. Stevenson R. Stone W. Wallace M. (2013). Multisensory speech perception in children with autism Spectrum disorder. J. Autism Child. Schizophr. 43, 2891–2902. doi: 10.1007/s10803-013-1836-5, PMID: 23624833 PMC3998667

[ref86] Young H. Oreve M. J. Speranza M. (2018). Clinical characteristics and problems diagnosing autism spectrum disorder in girls. Arch. Fr. Pediatr. 25, 399–403. doi: 10.1016/j.arcped.2018.06.008, PMID: 30143373

[ref87] Zander E. (2021). Social responsiveness scale. Second Edition (SRS-2) Edn Karolinska Institutet. Available at: https://ki.se/kind/social-responsiveness-scale-second-edition-srs-2

[ref88] Zhou H. Y. Cai X. L. Weigl M. Bang P. Cheung E. Chan R. (2018). Multisensory temporal binding window in autism spectrum disorders and schizophrenia spectrum disorders: a systematic review and meta-analysis. Neurosci. Biobehav. Rev. 86, 66–76. doi: 10.1016/j.neubiorev.2017.12.013, PMID: 29317216

[ref89] Zhou H. Y. Yang H. X. Shi L. J. Lui S. Cheung E. Chan R. (2021). Correlations between audiovisual temporal processing and sensory responsiveness in adolescents with autistic traits. J. Autism Dev. Disord. 51, 2450–2460. doi: 10.1007/s10803-020-04724-9, PMID: 32978707

